# Factors associated with response to compression-based physical therapy for secondary lower limb lymphedema after gynecologic cancer treatment: a multicenter retrospective study

**DOI:** 10.1186/s12885-021-09163-y

**Published:** 2022-01-03

**Authors:** Masato Yoshihara, Kaoru Kitamura, Satoko Tsuru, Ryoko Shimono, Hiromi Sakuda, Michinori Mayama, Sho Tano, Kaname Uno, Mayu Ohno Ukai, Yasuyuki Kishigami, Hidenori Oguchi, Akio Hirota

**Affiliations:** 1 Obstetrics and Gynecology, Nagoya University Graduate School of Medicine, 65 Tsuruma-cho, Showa-ku, Nagoya, Aichi 466-8550 Japan; 2 Obstetrics and Gynecology, TOYOTA Memorial Hospital, 1-1 Heiwa-cho, Toyota, Aichi 471-8513 Japan; 3 Department of Breast Surgery, Kaizuka Hospital, 7-7-27 Hakozaki, Higashi-ku, Fukuoka, Fukuoka, 812-0053 Japan; 4 School of Engineering, The University of Tokyo, 7-3-1 Hongo, Bunkyo-ku, Tokyo, 113-8656 Japan; 5 Organization for Interdisciplinary Research Project, The University of Tokyo, 7-3-1 Hongo, Bunkyo-ku, Tokyo, 113-8656 Japan; 6 Graduate School of Nursing, Osaka City University, 3-3-138, Sugimoto, Sumiyoshi-ku, Osaka-shi, 558-8585 Japan; 7 Obstetrics and Gynecology, Hokkaido University Graduate School of Medicine, Kita 8, Nishi 5, Kita-ku, Sapporo, Hokkaido 060-0808 Japan; 8 Hirota Internal Medicine Clinic, 5-19-10 Minami-karasuyama, Setagaya-ku, Tokyo, 157-0062 Japan

**Keywords:** Lymphedema, Gynecologic neoplasms, Prognosis, Compression bandage

## Abstract

**Background:**

Lower limb lymphedema (LLL) is one of the most refractory and debilitating complications related to gynecological cancer treatment. We investigated factors associated with response to compression-based physical therapy (CPT) for secondary LLL after gynecologic cancer treatment.

**Methods:**

We performed a multicenter retrospective study using the records of seven medical institutions from 2002 and 2014. Patients who developed LLL after gynecological cancer treatment were included. Limb volumes were calculated from the lengths of the limb circumferences at four points. All participants underwent compression-based physical therapy for LLL. Factors, including MLD, indicative of circumference reductions in LLL were determined.

**Results:**

In total, 1,034 LLL met the required criteria of for the study. A multivariate linear regression analysis identified age; body mass index (BMI); endometrial cancer; radiotherapy; and initial limb circumference as significant independent prognostic factors related to improvement in LLL. In analysis of covariance for improvement in LLL adjusted by the initial limb circumference and stratified by BMI and radiotherapy, patients with BMI 28 kg/m^2^ or higher and receiving radiation rarely responded to CPT.

**Conclusions:**

Improvements in the lower limb circumference correlated with clinical histories and physical characteristics, which may be used as independent prognostic factors for successful CPT for LLL after gynecological cancer treatment.

## Background

Lower limb lymphedema (LLL) is one of the most refractory and debilitating complications related to gynecological cancer treatment. The accumulation of lymphatic fluid in the limb interstitium caused by alterations in lymphatic flow due to surgery, chemotherapy, and radiation is the main cause of LLL [1, 2]. Its prevalence after gynecological cancer treatment widely ranges between 1 and 49%, with differences being attributed to the lack of standard diagnostic criteria [1]. Since LLL is a chronic and progressive disease, it ultimately reduces the physical and mental quality of life (QoL) of patients [3, 4].

A clinical history and physical examination are the most important elements for establishing a diagnosis of lymphedema. In the gynecological field, age, body mass index (BMI), type of cancer, radiotherapy, chemotherapy, and the number of removed lymph nodes have been identified as potential risk factors for LLL after gynecological cancer treatment [2, 3, 5–8]. In a physical examination, limb volume can be assessed by the water displacement method or estimated by taking several limb circumferential measurements at standard distance. It is important to evaluate serial changes in limb volumes in order to estimate the effects of lymphedema therapy [9–11].

The goal of lymphedema therapy is to maintain physical function, reduce psychological distress, and prevent development of infection. Initial therapy needs to be performed before extensive irreversible fibrosclerotic changes occur in the interstitium [12]. Compression-based physical therapy (CPT), such as banding and compression garments, are the most common and accessible approaches. The pressure induced by muscle contraction associated with banding or garments is considered to reduce lymphedema by mechanical stimulation of the smooth muscle of lymphatic vessels, which increases lymphatic flow [13]. The efficacy of CPT has been reported in patients with secondary upper limb lymphedema (ULL) and those with LLL [14]. Manual lymphatic drainage (MLD), which is a gentle manual technique that reroutes lymph flow around blocked areas into healthy lymph vessels and the venous system, is also one of the most common conservative therapies and is performed by specially trained physical therapists [11, 15]. The efficacy of MLD has been demonstrated in observational studies and small randomized trials; however, some studies have contraindicated its additive effects, the benefits of which remain unclear [10, 16–18].

Few studies have identified prognostic factors, including MLD, for LLL after gynecological cancer treatment. Alternatively, previous studies, including randomized control studies and systematic reviews, reported the positive effects of exercise, weight control, and physiotherapy for patients with ULL related to breast cancer treatment [19, 20]. Despite the differences in the characteristics of the causative disease, the pathogenesis of LLL is similar to that of ULL. Moreover, the identification of prognostic factors of LLL will enable estimates of the effects of LLL therapy and identify patients at high risk of treatment failure. Therefore, the aim of the present study was to identify factors associated with responses to CPT for secondary LLL after gynecological cancer treatment in a large population.

## Methods

### Study articipants

We conducted a multicenter retrospective study using the records of the following medical institutions from between April 2002 and November 2014: Hirota Internal Medicine Clinic, TOYOTA Memorial Hospital, Northern Fukushima Medical Center, Nagumo Clinic Fukuoka, Iwate Prefectural Miyako Hospital, Limbs Tokushima Clinic, and the Cancer Institute Hospital of the Japanese Foundation for Cancer Research. All of the institutions provide limb lymphedema therapy performed by a physician, nurse, physical therapist, and occupational therapist who completed 135 h of the coursework recommended in the statement of the National Lymphedema Network [15]. The present study was authorized and approved by the Ethical Committee of the School of Engineering of the University of Tokyo and performed in accordance with the principles of the Declaration of Helsinki.

Patients who developed LLL after gynecological cancer treatment were included in the present study. LLL was diagnosed based on physical findings and the International Society of Lymphology (ISL) staging system [10]. Some patients had bilateral LLL, the onset and extent of which varied; therefore, every affected limb was regarded as one sample number. We focused on the three main types of gynecological cancer (cervical, endometrial, and ovarian cancer) and excluded rare types, such as vulvar and vaginal cancer and Paget’s disease. LLL caused by definite diagnoses of primary heart failure, liver failure, and renal failure were also excluded.

### Data collection

We collected data on baseline characteristics, including age, BMI, the cancer type, site of lymphadenectomy (pelvic and pelvic with para-aorta), the performance of chemotherapy and radiotherapy, and MLD. Limb volumes were estimated by the total summed length of four axial limb circumferences taken at the ankle, crus, thigh, and groin areas. The measurements were performed on (1) the day on which the patient was diagnosed with LLL, and (2) approximately 4 to 7 weeks, and (3) 8 to 24 weeks after the initiation of LLL therapy and were defined as the (1) initial status, (2) early phase, and (3) maintenance phase, respectively. Data were collected by reviewing the charts of eligible patients and treating therapists measured the limb circumference of LLL.

### Statistical analysis

In univariate analysis, the relationship between each continuous variables (age, BMI, and limb circumference) and changes in limb circumference from the initial status to the early and maintenance phases were assessed by a linear regression analysis. On the other hand, for categorical variables (the cancer type, type of lymphadenectomy, chemotherapy, radiotherapy, stage, and MLD), change of limb circumference from the initial status to the early and maintenance phases were assessed by Student’s *t*-test. A multivariate regression analysis was performed to detect prognostic factors influencing reductions in limb circumference and included age, BMI, the cancer type, lymphadenectomy, chemotherapy, radiotherapy, the ISL stage, and initial limb circumference (the forced entry method). Changes in the limb circumference from initial status to the early and maintenance phases were evaluated by a repeated measures analysis of variance (ANOVA) with the post-hoc Bonferroni test. Differences in changes in the limb circumference from the initial status to the early and maintenance phases stratified by MLD, BMI (cutoff value of 28 kg/m^2^) and radiotherapy, were also estimated by a repeated measures analysis of covariance (ANCOVA) adjusted for baseline data as a covariate with the post-hoc Bonferroni test. Missing values were excluded in each analysis. The significance of differences was confirmed by two-sided P values, with the significance level set to P < 0.05. Statistical analyses were conducted with IBM SPSS Statistics for Windows, Version 25.0 (IBM Corp., Armonk, NY, USA).

## Results

### Baseline characteristics of the patients

In total, 1,034 LLL treated in one of the institutions during the study period, fulfilled the criteria of the present study. The baseline characteristics of the cases are listed in Table 1. There were 414 (40.0%), 274 (26.5%), and 159 (15.4%) cases of cervical, endometrial, and ovarian cancers, respectively. The remaining 187 (18.1%) cancer cases were recorded as uterine cancer of unknown origin. Pelvic lymphadenectomy was performed on 581 (56.2%) LLL cases, and additive para-aortic lymphadenectomy on 264 (25.5%). Eighty-seven (8.4%) LLL cases received chemotherapy, while 268 (25.9%) received radiotherapy. All cases received CPT, with MLD being performed on 881 (85.2%).

**Table 1:** Baseline characteristics of the patients with lower limb edema (*n* = 1,034)

**Characteristics**	**Category**		
**Age, year (SD)**		58.0	(11.7)
**BMI, kg/m**^**2**^** (SD)**		22.7	(3.9)
**Type of cancer, n (%)**	Cervical cancer	414	(40.0)
	Endometrial cancer	274	(26.5)
	Ovarian cancer	159	(15.4)
	Missing (Uterine cancer)	187	(18.1)
**Lymphadenectomy, n (%)**	Pelvic	581	(56.2)
	Pelvic + paraaorta	264	(25.5)
	Missing	189	(18.3)
**Chemotherapy, n (%)**		87	(8.4)
**Radiotherapy, n (%)**		268	(25.9)
**Stage, n (%)**	I	24	(2.3)
	II	885	(85.6)
	Late in II	109	(10.5)
	III	16	(1.5)
**Circumference, cm (SD)**	Total	163.9	(21.1)
	Ankle	23.1	(7.0)
	Crus	37.4	(5.9)
	Thigh	47.3	(6.3)
	Groin	56.0	(6.6)
**MLD, n (%)**		881	(85.2)

*SD* standard deviation, *BMI* body mass index, *MLD* manual lymphatic drainage

### Factors associated with response to CPT

We investigated factors associated with a good response to CPT for LLL among the background variables. In the multivariate analysis of factors associated with response to CPT in for LLL, four variables independently affected reductions in the total circumference from the initial status (the day of diagnosis) to the early phase (4 to 7 weeks after diagnosis): age, BMI, and initial circumference. In the maintenance phase (8 to 24 weeks after diagnosis), five variables were identified as independent prognostic factors for improvements in LLL from the initial status: age, BMI, endometrial cancer, radiotherapy, and initial circumference. On the other hand, MLD did not significantly affect changes in limb circumference in either phase. These results indicated that older age, lower BMI, cervical and ovarian cancer (compared to endometrial cancer), a larger initial limb circumference at diagnosis, and no radiotherapy were independently associated with greater reductions in limb circumference with CPT. (Table 2).

**Table 2:** Univariate and multiple regression analysis for changes of lower limb circumference

		**Early phase**				**Maintenance phase**			
		**Univariate analysis**		**Multivariate regression analysis**		**Univariate analysis**		**Multivariate regression analysis**	
**Characteristics**	**Categories**		***P* value**	**Standardized Coefficient**	***P* value**		***P* value**	**Standardized Coefficient**	***P* value**
**Age, CC**^**a**^		− 0.073	0.061	− 0.111	**0.007**	− 0.098	0.010	− 0.134	** < 0.001**
**BMI, CC**^**a**^		− 0.299	** < 0.001**	0.447	** < 0.001**	− 0.348	** < 0.001**	0.456	** < 0.001**
**Type of cancer, cm (SD)**^**b**^	Cervical cancer	− 7.20 (7.53)	0.268	referent		− 9.65 (9.64)	**0.008**	referent	
	Endometrial cancer	− 6.26 (7.01)		0.034	0.444	− 7.40 (9.64)		0.087	**0.037**
	Ovarian cancer	− 6.27 (7.37)		0.014	0.755	− 7.28 (8.81)		0.029	0.484
**Lymphadenectomy, cm (SD)**^**b**^	Pelvic	− 4.45 (9.90)	**0.014**	referent		− 8.46 (12.05)	** < 0.001**	referent	
	Pelvic + paraaorta	− 6.57 (8.17)		− 0.022	0.596	− 4.78 (9.29)		− 0.055	0.132
**Chemotherapy, cm (SD)**^**b**^	Yes	− 2.88 (10.66)	**0.002**	0.011	0.825	− 1.75 (11.24)	** < 0.001**	− 0.010	0.140
	No	− 6.88 (9.24)				− 8.29 (8.28)			
**Radiotherapy, cm (SD)**^**b**^	Yes	− 7.28 (12.29)	0.242	0.108	**0.012**	− 9.11 (14.53)	0.074	0.082	**0.040**
	No	− 6.35 (8.22)				− 7.16 (9.65)			
**Stage, cm (SD)**^**b**^	I	− 1.53 (5.32)	** < 0.001**	referent		− 0.13 (4.66)	** < 0.001**	referent	
	II	− 5.81 (7.61)		0.044	0.704	− 6.97 (9.32)		− 0.168	0.141
	Late in II	− 9.61 (10.57)		0.053	0.640	− 11.96 (12.65)		− 0.093	0.407
	III	− 27.32 (31.34)		0.01	0.849	− 31.76 (38.43)		− 0.056	0.260
**Circumference, CC**^**a**^		− 0.608	** < 0.001**	− 0.828	** < 0.001**	− 0.671	** < 0.001**	− 0.920	** < 0.001**
**MLD, cm (SD)**^**b**^	Yes	− 6.54 (9.35)	0.768	0.013	0.794	− 8.17 (11.52)	**0.012**	0.011	0.796
	No	− 6.87 (9.40)				− 5.40 (8.60)			

Statistically significant results are marked in bold

*BMI* Body mass index, *MLD* Manual lymphatic drainage

^a^Data are expressed as a correlation coefficient (CC)

^b^Data are expressed as the group mean (standard deviation [SD])

### Changes in Lower Limb Circumferences

**Fig. 1:**
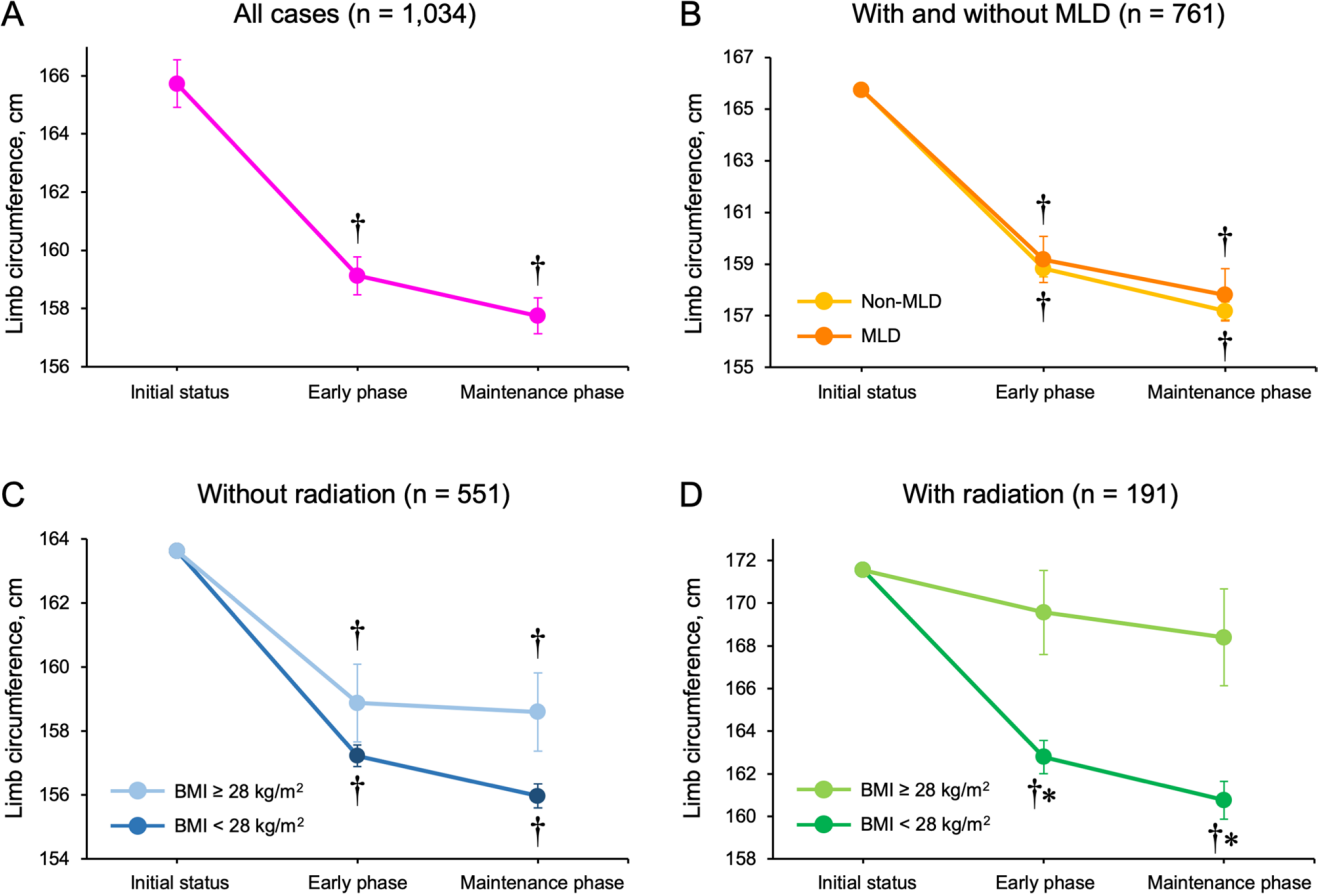
In comparisons with baseline values, limb circumference significantly decreased with compression therapy in the early and maintenance phases. **A** Comparisons of limb circumference variations between patients with and without manual lymphatic drainage (MLD). **B** Comparisons of limb circumference variations between patients with high BMI (≥ 28 kg/m^2^) and other patients divided based on the presence or absence of radiation therapy (**C** and **D**) *P < 0.05 significantly different from the other group. †*P* < 0.05 significantly different from the baseline

Based on the results in the multivariate regression analysis, we focused on BMI and radiotherapy as variables affecting responses to CPT and visualized the impact with statistical stratification. The initial limb circumference, one of the main factors affecting improvements in LLL, was adjusted with ANCOVA. Sequential changes in the lower limb circumference in the initial status (the day of diagnosis) and early (4 weeks to 7 weeks after diagnosis) and maintenance (8 weeks to 24 weeks after diagnosis) phases were examined (Fig. 1). In comparisons with baseline values, limb circumference significantly decreased with CPT in both the early and maintenance phases (Fig. 1A). Regarding therapeutic effects, MLD did not significantly affect changes in the limb circumference in the early and maintenance phases (Fig. 1B). Comparisons of limb circumference variations between patients with high BMI (≥ 28 kg/m^2^) and other patients who were divided according to whether radiation therapy was conducted are shown in Fig. 1C and D. In the cohort without radiotherapy, reductions in limb circumferences were similar in patients with BMI ≥ 28 kg/m^2^ and < 28 kg/m^2^ (Fig. 1C). However, in the cohort with radiotherapy, limb circumference was significantly shorter in patients with BMI < 28 kg/m^2^ than with ≥ 28 kg/m^2^ (Fig. 1D).

## Discussion

CPT exerted positive effects on LLL caused by gynecological cancer treatment. Although the intervals between the onset of LLL and treatment initiation were not evaluated in the present study, conventional physical therapeutic intervention achieved beneficial clinical outcomes for LLL. On the other hand, in the multivariate analysis, MLD was not a significant prognostic factor for improvements in LLL in the early or maintenance phase of treatment. A literature review reported both positive and ambiguous findings on the efficacy of MLD for lymphedema [10, 16–18]; however, MLD is a common technique for patients with ISL class II in Japan, and its efficacy and potential applications need to be further examined in more detail in future studies including prospective trials.

The multivariate regression analysis also identified independent prognostic factors associated with a good response to CPT for LLL. The initial limb circumference was identified as the most influential positive prognostic factor for improvements in LLL, which can be explained by the larger accumulation of leaked lymphatic fluid in the interstitium; this fluid was easily transported by the drainage effect of compression, as previously reported [21]. Age also had a significant positive effect for greater reductions of lower limb circumference. In clinical settings, the deterioration of LLL is common in patients who cannot focus on LLL therapy and are forced to keep standing up because of their jobs or housekeeping. In addition, elderly patients are more likely to have hypertension or disuse syndrome, and some may be taking edema-inducing drugs [22], the effects of which may achieve superficial improvements in curable non-lymphatic edemas. On the other hand, higher BMI, radiation therapy, and endometrial cancer decreased the range of reductions in limb circumference with CPT. Obesity impairs natural lymphatic flow and its drainage system, which is one of the direct causes of LLL [23]. It has been identified as one of the risk factors for the development of ULL and LLL after cancer treatment [3, 24]. Although weight control during the treatment course was not examined in the present study, it is considered to play a key role in the amelioration of this cancer-associated comorbidity. Radiotherapy also had a negative impact on improvements in LLL, and was also previously reported to affect the incidence of LLL [25]. In ULL associated with breast cancer treatment, radiotherapy is one of the main risk factors contributing to the development and progression of ULL [26], which can also aggravate lymphedema in lower limbs. In ANCOVA stratified by the presence and absence of radiotherapy, a significant difference was observed in treatment effects between patients with high BMI (≥ 28 kg/m^2^) and other patients. Therefore, obese patients with radiotherapy may require more careful management and active treatment for LLL, including lymphaticovenular anastomosis [27]. Endometrial cancer was identified as an independent risk factor for the interference of LLL therapy in the maintenance phase; however, the underlying reason was unclear. Some confounding factors related to the development of endometrial cancer, that are not reflected by BMI, such as diabetes mellitus and adiposis [28, 29], which were not investigated, may have contributed to this result; therefore, further studies are warranted. Other factors, including the site of lymphadenectomy, chemotherapy, and ISL stage, were not significant independent predictors of improvements in LLL in the multivariate analysis in this study population.

The limitations of the present study include changes in the QoL of patients not being examined. Lymphedema is an incurable disease that affects esthetics and restricts the activities of daily living of affected patients, such as dressing and movement. These factors have been associated with psychological reductions in the QoL of patients with cancer-related ULL and LLL [3, 4]. It was also unclear whether self-management for LLL in daily life, such as exercise, weight control, and skin care, influenced the outcome Due to the difficulties associated with extrapolating this retrospective analysis directly to any recommendations for clinical practice, the present results need to be primarily used as the basis for additional prospective studies.

## Conclusion

Improvements in the lower limb circumference correlated with clinical histories and physical characteristics, which may be used as independent prognostic factors for successful CPT for LLL after gynecological cancer treatment.

## Data Availability

The data that support the results of the present study are available from the University of Tokyo; however, restrictions apply to the availability of these data, which were used under license for the present study, and, thus, are not publicly available. Data are available from the authors upon reasonable request and with permission by the University of Tokyo.

## Abbreviations

LLL: Lower limb lymphedema
QoL: Quality of life
BMI: Body mass index
ULL: Upper limb lymphedema
MLD: Manual lymphatic drainage
ISL: International Society of Lymphology
ANOVA: Analyzed by one-way analysis of variance

## References

[1] Beesley V, Janda M, Eakin E, Obermair A, Battistutta D (2007). Lymphedema after gynecological cancer treatment. prevalence, correlates, and supportive care needs. Cancer.

[2] Hareyama H, Hada K, Goto K (2015). Prevalence, classification, and risk factors for postoperative lower extremity lymphedema in women with gynecologic malignancies: a retrospective study. Int J Gynecol Cancer.

[3] Yost KJ, Cheville AL, Al-Hilli MM (2014). Lymphedema after surgery for endometrial cancer: prevalence, risk factors, and quality of life. Obstet Gynecol.

[4] Rowlands IJ, Beesley VL, Janda M (2014). Quality of life of women with lower limb swelling or lymphedema 3–5 years following endometrial cancer. Gynecol Oncol.

[5] Beesley VL, Rowlands IJ, Hayes SC (2015). Incidence, risk factors and estimates of a woman’s risk of developing secondary lower limb lymphedema and lymphedema-specific supportive care needs in women treated for endometrial cancer. Gynecol Oncol.

[6] Abu-Rustum NR, Alektiar K, Iasonos A (2006). The incidence of symptomatic lower-extremity lymphedema following treatment of uterine corpus malignancies: a 12-year experience at Memorial Sloan-Kettering Cancer Center. Gynecol Oncol.

[7] Deura I, Shimada M, Hirashita K (2015). Incidence and risk factors for lower limb lymphedema after gynecological cancer surgery with initiation of periodic complex decongestive physiotherapy. Int J Clin Oncol.

[8] Graf N, Rufibach K, Schmidt AM, Fehr M, Fink D, Baege AC (2013). Frequency and risk factors of lower limb lymphedema following lymphadenectomy in patients with gynecological malignancies. Eur J Gynaecol Oncol.

[9] Chen YW, Tsai HJ, Hung HC, Tsauo JY (2008). Reliability study of measurements for lymphedema in breast cancer patients. Am J Phys Med Rehabil.

[10] International Society of Lymphology (2013). The diagnosis and treatment of peripheral lymphedema: 2013 Consensus Document of the International Society of Lymphology. Lymphology.

[11] Zuther JE (2009). Complete Decongestive Therapy: Lymphedema Management -The Comprehensive Guide for Practitioners.

[12] Mayrovitz HN (2009). The standard of care for lymphedema: current concepts and physiological considerations. Lymphat Res Biol.

[13] Lawenda BD, Mondry TE, Johnstone PA (2009). Lymphedema: a primer on the identification and management of chronic condition in analogic treatment. CA Cancer J Clin.

[14] Lasinski BB, McKillip Thrift K, Squire D (2012). A systematic review of the evidence for complete decongestive therapy in the treatment of lymphedema from 2004 to 2011. PM & R.

[15] National Lymphedema Network. Position Paper: Training of Lymphedema Therapists. Available from URL: http://www.lymphnet.org/resources/position-paper-training-of-lymphedema-therapists. Accessed 1 Jan 2016.

[16] Williams AF, Vadgama A, Franks PJ, Mortimer PS (2002). A randomized controlled crossover study of MLD therapy in women with breast cancer-related lymphedema. Eur J Cancer Care.

[17] Huang TW, Tseng SH, Lin CC (2013). Effects of manual lymphatic drainage on breast cancer-related lymphedema: a systematic review and meta-analysis of randomized controlled trials. World J Surg Oncol.

[18] Johansson K, Karlsson K, Nikolaidis P (2015). Evidence-based or traditional treatment of cancer-related lymphedema. Lymphology.

[19] De Groef A, Van Kampen M, Dieltjens E (2015). Effectiveness of postoperative physical therapy for upper-limb impairments after breast cancer treatment: a systematic review. Arch Phys Med Rehabil.

[20] Shaw C, Mortimer P, Judd PA (2007). A randomized controlled trial of weight reduction as a treatment for breast cancer-related lymphedema. Cancer.

[21] Liao SF, Li SH, Huang HY (2012). The efficacy of complex decongestive physiotherapy (CDP) and predictive factors of response to CDP in lower limb lymphedema (LLL) after pelvic cancer treatment. Gynecol Oncol.

[22] Braunwald E, Loscalzo J, Fauci AS, Braunwald E, Kasper DL (2008). Edema. Harrison’s principles of internal medicine.

[23] Greene AK, Grant FD, Slavin SA (2012). Lower-extremity lymphedema and elevated body-mass index. N Engl J Med.

[24] Helyer LK, Varnic M, Le LW, Leong W, McCready D (2010). Obesity is a risk factor for developing postoperative lymphedema in breast cancer patients. Breast J.

[25] Yoshihara M, Shimono R, Tsuru S (2020). Risk factors for late-onset lower limb lymphedema after gynecological cancer treatment: A multi-institutional retrospective study. Eur J Surg Oncol.

[26] Warren LE, Miller CL, Horick N (2014). The impact of radiation therapy on the risk of lymphedema after treatment for breast cancer: a prospective cohort study. Int J Radiat Oncol Biol Phys.

[27] Campisi C, Bellini C, Campisi C, Accogli S, Bonioli E, Boccardo F (2010). Microsurgery for lymphedema: clinical research and long-term results. Microsurgery.

[28] Aune D, Navarro Rosenblatt DA, Chan DS (2015). Anthropometric factors and endometrial cancer risk: a systematic review and dose-response meta-analysis of prospective studies. Ann Oncol.

[29] Liao C, Zhang D, Mungo C, Tompkins DA, Zeidan AM (2014). Is diabetes mellitus associated with increased incidence and disease-specific mortality in endometrial cancer? A systematic review and meta-analysis of cohort studies. Gynecol Oncol.

